# Supplementary material for: A fault-intrusion-tolerant system and deadline-aware algorithm for scheduling scientific workflow in the cloud
Source: PeerJ Comput Sci. 2021 Nov 2;7:e747. doi: 10.7717/peerj-cs.747 (PMC8576554; doi:10.7717/peerj-cs.747)
Supplement: Supplemental Information 6 [file peerj-cs-07-747-s006.zip › Myproject1.1/docs/org/cloudbus/cloudsim/examples/power/random/Dvfs.html]

Dvfs


JavaScript is disabled on your browser.


- Overview
- Package
- Class
- Use
- Tree
- Deprecated
- Index
- Help

- Prev Class
- Next Class

- Frames
- No Frames

- All Classes

- Summary:
- Nested |
- Field |
- Constr |
- Method

- Detail:
- Field |
- Constr |
- Method


org.cloudbus.cloudsim.examples.power.random

## Class Dvfs

- java.lang.Object
- - org.cloudbus.cloudsim.examples.power.random.Dvfs

- ---

    

  ```
  public class Dvfs
  extends java.lang.Object
  ```

  A simulation of a heterogeneous power aware data center that only applied DVFS, but no dynamic
  optimization of the VM allocation. The adjustment of the hosts' power consumption according to
  their CPU utilization is happening in the PowerDatacenter class.
  The remaining configuration parameters are in the Constants and RandomConstants classes.
  If you are using any algorithms, policies or workload included in the power package please cite
  the following paper:
  Anton Beloglazov, and Rajkumar Buyya, "Optimal Online Deterministic Algorithms and Adaptive
  Heuristics for Energy and Performance Efficient Dynamic Consolidation of Virtual Machines in
  Cloud Data Centers", Concurrency and Computation: Practice and Experience (CCPE), Volume 24,
  Issue 13, Pages: 1397-1420, John Wiley & Sons, Ltd, New York, USA, 2012

  Since:
  :   Jan 5, 2012

- - ### Constructor Summary

    Constructors

    Constructor and Description

    `Dvfs()`
  - ### Method Summary

    Methods

    | Modifier and Type | Method and Description |
    | --- | --- |
    | `static void` | `main(java.lang.String[] args)` The main method. |

    - ### Methods inherited from class java.lang.Object

      `clone, equals, finalize, getClass, hashCode, notify, notifyAll, toString, wait, wait, wait`

- - ### Constructor Detail


    - #### Dvfs

      ```
      public Dvfs()
      ```
  - ### Method Detail


    - #### main

      ```
      public static void main(java.lang.String[] args)
                       throws java.io.IOException
      ```

      The main method.

      Parameters:
      :   `args` - the arguments

      Throws:
      :   `java.io.IOException` - Signals that an I/O exception has occurred.


- Overview
- Package
- Class
- Use
- Tree
- Deprecated
- Index
- Help

- Prev Class
- Next Class

- Frames
- No Frames

- All Classes

- Summary:
- Nested |
- Field |
- Constr |
- Method

- Detail:
- Field |
- Constr |
- Method
